# The Evolution and Abuse of the Serration

**Published:** 1897-03

**Authors:** 


### Editorial.

### THE EVOLUTION AND ABUSE OF THE SERRATION.

THE formation and development of a profession or calling is always an interesting subject for the historian, and for those specially trained in this direction there is no study more productive of good results. The busy worker performing his daily task, although a part of the machinery of evolution, has no time, or is apt to think he lacks leisure sufficient, to go beyond the present. That this is a mistake and leads to serious and amusing blunders needs no assertion, the proof being furnished in almost every dental periodical. That a few have made an attempt to rescue the almost forgotten work of the past and forced it to yield up its treasures, even in a limited degree, is a very satisfactory indication that the thought is growing that progress cannot be intelligently made unless the steps leading to the point attained be carefully and reverently trodden.

Minor details, apparently insignificant, but of vital importance when viewed in their connections, are being daily consigned to oblivion, and the patient investigator of historic problems is forced to say that the origin of this or that is lost in the obscurity of the past.

It, therefore, becomes the duty of the faithful chronicler to occasionally pause and permit the varied problems of the present to pass by, or be solved by others, while he brings to the surface some of the apparently insignificant things in the profession of dentistry.

With something of this spirit the writer would call attention to the evolution of the serration and its use, progress, and, it might be added, the abuse in the work of compacting metals in teeth.

The history of the serration is quite modern, as it is understood and connected with dentistry. The writer has been unable to find much in the literature of this profession in relation to it prior to the middle of the present century. In the edition of 1850 of Harris's "Principles and Practice," no allusion is made to serrations, but his instruments for condensing gold show a simple crucial file-mark on the edges. This corresponds with the memory of the writer, for at that time the serration, as now understood, was unknown, and dentists very frequently made the cross cut with the file on their smooth-pointed instruments. It is also within the memory of the writer that rounded and flat points were used almost exclusively to pack the soft gold in the early forties.

From 1850 to the introduction of cohesive gold, it is very certain that the dental profession, as a whole, found very little use for the serration in the condensing of non-cohesive gold, and operators were content with a roughness made, as heretofore stated, by hand-filing, with no attempt to make these a part of the process of condensation.

Those who were in practice when Dr. Arthur introduced cohesive gold in 1855 will, doubtless, recall the frequent abortive efforts to use this gold with the instruments then provided. Filling by the use of this character of foil, which is the character all gold possesses when not tampered with after refining, if not impossible, was at least rendered very difficult, and many never succeeded in filling teeth with it.

The explanation for this was to be found in the fact that the points of pluggers were poorly fitted for the work. It then became a necessity to have serrations, and from this date, 1855, it must be conceded, the serration took its rise. The first made were but little more than exaggerations of the crucial file cut; but eventually they assumed various forms, some almost straight, needle-shaped, and others, triangular or pyramidal, extending from a common base, and all, of whatever form, exceedingly sharp. This latter quality was considered essential, so much so, that the manufacturers furnished knife-edged stones to enable the dentist to keep these serrations always up to the original standard.

Then came the introduction of the mallet in its protean forms, hand, automatic, and power, and then it was discovered that the older, sharp serration was not only unnecessary, but was regarded as an absolute injury by pitting the gold. This led to a gradual reduction of the serrations until we have instruments to-day on sale, at the various supply-houses, that are, in the opinion of the writer, practically unfit to use in filling teeth. The circle has been thoroughly traversed, and from the original file cut we have come round again to very nearly the same form, having tried all the intermediate stages of serrations.

The question that interests us to-day is, Has this movement in the circle been of advantage to the profession of dentistry in its practical work? In order to answer this query intelligently the object of the serration must first be considered; originally it was nothing more than a means to prevent the instrument slipping over the gold, and in no sense could it be regarded as a factor in condensation. With the use of cohesive foil it was found that the cohesive property could not be relied upon universally. Theoretically it was true that two clean surfaces of gold must cause cohesion through the attraction of the atoms, alike common to solids and fluids, so that two sheets of gold could not be separated when fairly placed one upon the other. If this property could always have been maintained, then filling of a tooth simply meant adding layer upon layer, but it was soon discovered that this did not always occur, and the dentist of the period was obliged not only to remove this tendency to non-cohesiveness by heat, but also was forced to use some means to insure the union of each lamina of foil, hence the sharp serration which accomplished it in part mechanically, by pinning one piece to another. This was found not quite so essential under the direct impact of the mallet, and, as heretefore stated, serrations of the sharp variety were generally abandoned.

If gold foil could be maintained at the same standard as it leaves the hands of the manufacturers, and that indefinitely, there would be no need of serrations. This, however, is not the case with gold foil, for the reason, demonstrated by Dr. Black, that gold is peculiarly liable to be affected by gaseous deposits, which largely destroy its cohesive property. The impossibility of securing fresh gold foil by the great majority of dentists must ever make this form of cohesive gold a source of difficulty to many operators, and especially so to beginners. Annealing may help, but it does not entirely remove the objection, neither does the remedial process proposed by Black, and the fact remains that, to the man who works a comparatively small amount of gold foil, its use, with the present form of serrated instrument, has become a matter of serious concern, and this will continue just as long as manufacturers depend for their information, of what is needed, upon the opinion of those nearest the centre of professional excellence.

It is quite evident to some of those who are called to observe the struggles of beginners that the serrations of to-day are practically wrong, and while this may not be so serious with power mallets and gold, it becomes an absolute bar to good work with hand pressure and with tin. This metal must be used, if used at all, with a proper understanding of its cohesive property. The serrations here must be of a positive character and sharply defined.

The theory of some that gold can be burnished into a cavity by a smooth point or a point simply roughened is true only in a modified sense. That a satisfactory filling can be placed in by this method there can be no question, but is it of universal application? The answer, it would seem to the writer, must be in the negative. With the daily deterioration of gold foil after it leaves the manufacturer, the necessity for deeper serrations is evident, and the further removed the individual is from the great centres of supply the more will these be required.

The argument used against deep serrations is, as before stated, that these pit the gold. This is not a serious objection and need not hold with the last layers, which can be condensed by broader instruments and shallower serrations.

It has been very evident to many that the standard of work in filling has greatly degenerated in the past few years. Formerly the class of superior operators was the rule and the inferior the exception, but conditions are exactly the reverse. If this be true, and there are good reasons for the opinion, the cause of the falling off should be investigated. It is particularly noticeable in the rising generation of dentists, and it is the decided opinion of the writer that this may be largely ascribed to erroneous ideas, prevailing generally in regard to instruments as well, in some degree, to methods.

The intention of this article will have been subserved if it succeeds in calling attention to the necessity of reviewing this whole subject. It is a delicate matter and stands on debatable ground, for those wedded to different methods will set their faces sternly against the views here promulgated, and, perhaps, with good reason, for they may never have had occasion to work with gold foil, months from the gold-beater's hands. When they have had this experience, they will learn that while all is "not gold that glitters," neither is all gold cohesive that goes under that name. In order to use it without fear and without embarrassment, there must be a return, not to the needle-shapes of the earlier serrations, but to the common sense form that will aid in interlocking the crystals without an entire dependence on the unstable property of cohesion.

